# Prognostic implications of *Fibroblast growth factor receptor 1 (FGFR1)* gene amplification and protein overexpression in hypopharyngeal and laryngeal squamous cell carcinoma

**DOI:** 10.1186/s12885-020-06792-7

**Published:** 2020-04-18

**Authors:** Eun Kyung Kim, Yoon Ah Cho, Yoon Woo Koh, Hyang Ae Shin, Byoung Chul Cho, Sun Och Yoon

**Affiliations:** 1 Department of Pathology, National Health Insurance Service Ilsan Hospital, Goyang, 10444 South Korea; 2 Department of Pathology, Severance Hospital, Yonsei University College of Medicine, 50-1 Yonsei-ro, Seodaemun-gu, Seoul, 03722 South Korea; 3 Department of Pathology and Translational genomics, Samsung Medical Center, Seoul, 06351 South Korea; 4 Department of Otorhinolaryngology, Severance Hospital, Yonsei University College of Medicine, Seoul, 03722 South Korea; 5 Department of Otorhinolaryngology-Head Neck Surgery, National Health Insurance Service Ilsan Hospital, Goyang, 10444 South Korea; 6 Division of Medical Oncology, Yonsei Cancer Center, Yonsei University College of Medicine, 50-1 Yonsei-ro, Seodaemun-gu, Seoul, 03722 South Korea

**Keywords:** Fibroblast growth factor receptor 1 *(FGFR1)*, Amplification, Fluorescence in situ hybridization, Immunohistochemistry, Hypopharynx, Larynx, Squamous cell carcinoma

## Abstract

**Background:**

The gene encoding fibroblast growth factor receptor 1 (*FGFR1*) is emerging as a therapeutic and prognostic biomarker in various cancer types, including head and neck squamous cell carcinoma (SCC). Here, we investigated the clinicopathologic implication of *FGFR1* gene amplification and protein overexpression in hypopharyngeal and laryngeal SCC.

**Methods:**

Fluorescence in situ hybridization and immunohistochemistry were performed to determine FGFR1 gene amplification and protein overexpression in 209 surgically resected cases.

**Results:**

*FGFR1* amplification observed in 8 (8/66, 12.1%; 6 hypopharynx and 2 larynx) patients and high FGFR1 expression in 21 (21/199, 10.6%) patients significantly correlated with lymph node metastasis and advanced pathological stages. *FGFR1* amplification was also associated with worse disease-free survival in multivariate analysis (hazard ratio = 4.527, *P* = 0.032). High FGFR1 expression was more frequently observed, consistent with the worsening of the degree of histologic differentiation.

**Conclusions:**

*FGFR1* amplification may serve as an independent prognostic factor for disease-free survival in hypopharyngeal and laryngeal SCC. Aberrant FGFR signaling caused by *FGFR1* gene amplification or protein overexpression may play a crucial role in the malignant evolution and progression of hypopharyngeal and laryngeal SCC, and offer novel therapeutic opportunities in patients with hypopharyngeal and laryngeal SCC that usually lack specific therapeutic targets.

## Background

Head and neck squamous cell carcinoma (HNSCC) arises from the squamous mucosa of the upper aerodigestive tract, which comprises the nasal cavity, paranasal sinus, oral cavity, nasopharynx, oropharynx, hypopharynx, and larynx. These distinct anatomic subsites contribute to the morphological, biological, and etiological heterogeneities of HNSCC. Cigarette smoking and alcohol consumption are the most common risk factors, especially for hypopharyngeal and laryngeal SCC [1, 2], while infection with high-risk human papilloma virus (HPV) has also been recognized as a risk factor associated with oropharyngeal SCC [3]. Recent studies have shown that HPV-positive SCC, mainly oropharyngeal SCC, exhibits significantly favorable survival outcomes [4–6]. However, SCCs of the hypopharynx and larynx, the second most common respiratory tract cancer after lung cancer, that represent HPV-negative HNSCCs are related to little improvement in patient outcomes despite multidisciplinary treatments because of frequent locoregional recurrences, distant metastases, and second primary tumors [7, 8].

The Cancer Genome Atlas (TCGA) has recently identified potentially targetable somatic genomic alterations based on HPV infection status, smoking, and primary tumor sites in HNSCC [9]. HPV-negative tumors are characterized with recurrent focal amplifications in receptor tyrosine kinases and fibroblast growth factor receptor 1 (*FGFR1;* 10% frequency) is the second most commonly observed gene after *EGFR* (15% frequency). The gene encoding FGFR1 is located on chromosome 8p11.23 and encodes tyrosine kinase family, which plays crucial roles in cancer development. This gene is dysregulated by amplification, point mutation, translocation, and overexpression in various cancers [10]. These aberrant *FGFR1* alterations, in general, lead to gain-of-function characteristics and constitutively activate the downstream RAS/mitogen-activated protein kinase (MAPK), PI3K/protein kinase B (AKT), and Janus kinase (JAK)/signal transducer and activator of transcription (STAT) signaling pathways [11]. In previous studies of HNSCC, *FGFR1* amplification has been reported in 3 to 17% of cases, and FGFR1 protein overexpression has been identified in about 11–82% of cases [12–18]. However, they presented conflicting results for *FGFR1* as a prognostic biomarker. In addition, most of the studies have been conducted on the whole HNSCCs showing biological heterogeneity, and site-specific studies have been rarely performed on SCC, especially in SCCs of hypopharynx and larynx, which represent the prevalent subsites of HPV-negative SCC [13–19]. Therefore, more evidence is needed for the prognostic or predictive role of *FGFR1* in HNSCC of hypopharynx and larynx.

As a predictive marker for drug response, *FGFR1* has been identified in preclinical or clinical studies of lung SCC or breast cancer [19–21]. Recently, several nonselective or selective tyrosine kinase inhibitors suppressing FGFR1 expression, such as lucitanib (E3810), dovitinib (TKI258), ponatinib (AP24534), AZD4547, BGJ398, and TAS-120, have shown promising data or are currently being investigated in preclinical models and clinical trials on solid tumors, including HNSCC (NCT02706691, NCT02795156) [22, 23]. However, effective targeted therapies for advanced HNSCC are still limited to the anti-EGFR monoclonal antibody, cetuximab, in HNSCC [24].

In this study, we evaluated FGFR1 gene amplification and protein overexpression and investigated its clinicopathologic and prognostic implications in hypopharyngeal and laryngeal SCC.

## Methods

### Patients and tissue samples

Archived formalin-fixed, paraffin-embedded (FFPE) specimens were obtained from patients with surgically resected primary hypopharyngeal and laryngeal SCC. The surgical resections, such as traditional or transoral robotic laryngopharyngectomy, excision, and cordectomy, were performed at Severance Hospital, Seoul, South Korea and National Health Insurance Service Ilsan Hospital, Goyang, South Korea, between 2005 and 2012 for curative aim. From the consecutive cases, 209 cases were selected when tumor tissues, clinical data (including smoking status), and survival data were available; patients received no preoperative treatment, and no clinicopathological evidences of distant metastasis were reported at the time of surgery. Relapsed patients were excluded. After surgery, some patients received adjuvant treatment, such as chemotherapy, radiotherapy, and concurrent chemoradiation therapy, based on the National Comprehensive Cancer Network (NCCN) guidelines and clinical judgment.

Two pathologists (S.O.Y. and E.K.K.) evaluated the histologic features, including tumor location, size, grade, metastasis to regional lymph nodes, lymphovascular invasion, and perineural invasion, and confirmed the histopathologic diagnosis. Tumors were classified according to the eighth American Joint Committee on Cancer (AJCC) cancer system [25] and the World Health Organization system [2]. Clinical data were collected and reviewed as per patients’ medical records. Smoking status was defined as follows; “Never smokers” are those who have smoked less than 100 cigarettes in their lifetime, “former smokers” are those who have quit smoking for more than 12 months, and “current smokers” are those who are currently smoking or who have quit smoking for less than 12 months [26]. Alcohol consumption status was assessed as pure alcohol consumption, calculated as gram per day according to the average amount, frequency, and type. “Heavy drinkers” were those who consumed more than 30 g/day, and those who drank less were defined as “social drinkers” [27]. The study was approved by the Institutional Review Board of Severance Hospital (4–2015-0954) and National Health Insurance Service Ilsan Hospital (NHIMC 2018–04-021).

### Tissue microarray (TMA) construction

Sections of FFPE tissues were prepared and stained with hematoxylin and eosin. The representative areas of tumors were confirmed and selected to obtain a TMA under the microscope. One to three different representative areas per case were selected, and core tissues (3 mm in diameter) were obtained from the individual FFPE blocks. Considering the possibility of heterogeneity, randomly selected 35 cases were stained on whole section slides.

### *FGFR1* fluorescence in situ hybridization (FISH)

We conducted the FISH assay on the TMAs using commercially available *FGFR1* probes that hybridized to the 8p12–8p11.23 region using the fluorophore, Spectrum Orange (red) and to the centromere region of chromosome 8 (CEP 8) using the fluorophore, Spectrum Green (Abbott Molecular, Abbott Park, IL) following the manufacturers’ protocol. FISH results were interpreted by two expert evaluators (S.O.Y. and E.K.K.) without knowing the clinical information. Cells with sharp borders of nuclei, no signs of over-digestion, or non-overlapping nuclei were counted. Normal tissues including blood vessels, fibroblasts, or adjacent normal squamous epithelium served as internal positive controls. Tumor tissue was scanned for hot spots under 40× or 63× objective lens. Twenty contiguous tumor cell nuclei from three hot spots or random areas resulting in a total of 60 nuclei were individually evaluated under the 100× objective lens by counting red FGFR1 and green CEP8 signals. *FGFR1* gene amplification was defined as 1) an *FGFR1*/CEP8 ratio of at least 2 and the average number of *FGFR1* signals per tumor cell nucleus of at least 4 or 2) an average number of *FGFR1* signals per tumor cell nucleus of at least 6 [15, 28].

### FGFR1 immunohistochemistry (IHC)

FGFR1 protein expression was evaluated with IHC using a rabbit polyclonal anti-FGFR1 antibody (Clone ab10646, 1:1500 dilution, Abcam, Cambridge, UK) on 4-μm TMA tissue sections on a Ventana Bench Mark XT Autostainer (Ventana Medical Systems, Tucson, AZ, USA). FGFR1 staining pattern (cytoplasmic, membranous, or nuclear) and intensity (0, negative; 1, weak; 2, moderate; and 3, strong), and the percentage of positively stained tumor cells (0–100%) were evaluated. Staining pattern of normal squamous epithelial cells and stromal cells adjacent to or separated from tumors was compared to that of tumor cells. In addition, IHC was performed for Snail (dilution 1:200; Invitrogen, Thermo Fisher Scientific, CA, USA) and Twist (dilution 1:200; Abcam, Cambridge, UK), which are transcription factors related to epithelial mesenchymal transition (EMT). IHC expression of FGFR1, Snail, and Twist was analyzed using the semi-quantitative H-score method, which yields a possible score range of 0–300 obtained by multiplying the dominant intensity score with the percentage of positive tumor cells.

In total, 171 samples subjected to p16 IHC (a mouse monoclonal antibody, clone E0037, Ventana, AZ, USA) at the time of diagnosis were reviewed. p16 expression was scored as positive upon detection of at least 70% nuclear and cytoplasmic expression, with at least moderate to strong intensity [29].

### Statistical analysis

The chi-square, Fisher’s exact, independent-samples t-tests, and bivariate correlation analysis were conducted to compare the clinicopathologic parameters among patients with FGFR1 gene amplification and other protein expression. Statistical significance was set at *P* <  0.05 for all analyses. Patient survival rates were determined using the Kaplan-Meier method, and differences in survival rates were compared using the log-rank test. Disease-free survival (DFS) was measured from the time of surgery until disease progression, and was defined as cancer recurrence, continuance of progressive disease without complete remission, or cancer-related death. Overall survival (OS) was calculated from the date of surgery to the date of death or last follow-up visit. Multivariate analysis was performed with the Cox proportional hazard model using several clinicopathologic parameters. All statistical analyses were performed with IBM SPSS 22 software for Windows (IBM Corp, Somers, New York).

## Results

### Demographic characteristics of patients with hypopharyngeal and laryngeal SCC

The clinical and pathological characteristics of 209 patients are summarized in Table 1. A total of 195 (93.3%) patients were males and 14 (6.7%) were females, with a median age of 64 years (range 30–88 years). The cohort comprised 54 patients with (25.8%) hypopharyngeal SCC and 155 patients with (74.2%) laryngeal SCC. The majority of patients (*n* = 186, 89%) were current or former smokers, with a median smoking dosage of 30.0 pack-years (range 0–100). Furthermore, the majority of patients (*n* = 149, 87.1%) were heavy drinkers. Histological analysis revealed that most SCCs (*n* = 187, 89.5%) were well to moderately differentiated and revealed p16 IHC negativity (n = 149, 87.1%), as evident from 171 cases with available p16 IHC data. Advanced pT classification (pT3 and pT4a; *n* = 80, 38.3%) and advanced pN classification, especially pN3b (*n* = 52, 24.9%), were not uncommon. TNM stages were as follows: stage I in 28.7% cases, stage II in 13.9% cases, stage III in 15.3% cases, stage IVA in 17.2% cases, and stage IVB in 24.9% cases. Adjuvant treatment (concurrent chemoradiation therapy and radiation therapy) was provided to 120 (57.4%) patients. During the observation period with a median follow-up time of 38.8 months, 21.5% (*n* = 45) patients experienced recurrence. The mean DFS and OS rates for all patients were 82.3 months (range, 2–105 months) and 76.4 months (2–105 months), respectively. The 5-year DFS and OS rates were 74.1 and 67.6%, respectively.

**Table 1:** Demographic characteristics of hypopharynx and larynx squamous cell carcinoma

Characteristics	All patients n (%)	Characteristics	All patients n (%)
Total	209 (100)		
Age (years)		pT-classification	
Median (range)	64.0 (30–88)	pT1	71 (34.0)
< 65	114 (54.5)	pT2	58 (27.8)
≥ 65	95 (45.5)	pT3	52 (24.9)
Gender		pT4a	28 (13.4)
Female	14 (6.7)	pN-classification	
Male	195 (93.3)	pN0	118 (56.5)
Primary sites		pN1	13 (6.2)
Hypopharynx	54 (25.8)	pN2	26 (12.5)
Larynx	155 (74.2)	pN3	52 (24.9)
Smoking		Pathological stage	
Never smoker	23 (11.0)	Stage I	60 (28.7)
Former smoker	54 (25.8)	Stage II	29 (13.9)
Current smoker	132 (63.2)	Stage III	32 (15.3)
Alcohol		Stage IVA	36 (17.2)
Non- or social- drinker	45 (20.1)	Stage IVB	52 (24.9)
Heavy drinker	167 (79.9)	Adjuvant treatment	
Histologic differentiation		No	89 (42.6)
Well differentiated	64 (30.6)	Yes	120 (57.4)
Moderately differentiated	123 (58.9)	Chemotherapy (C)	0 (0.0)
Poorly differentiated	22 (10.5)	Radiation therapy (R)	71 (34.0)
Lymphovascular invasion		C + R	49 (23.4)
Absent	163 (78.0)	Recurrence	
Present	46 (22.0)	No	164 (78.5)
Perineural invasion		Yes	45 (21.5)
Absent	178 (85.2)	Local recurrence only	23 (51.1)
Present	31 (14.8)	Distant recurrence	22 (48.9)
Positive resection margin		Disease-free survival	
Absent	155 (74.2)	Mean (range; months)	82.3 (2–105)
Present	54 (25.8)	Overall survival time	
p16 status (*n* = 171)		Mean (range; months)	76.4 (2–105)
Negative	149 (87.1)		
Positive	22 (12.9)		

### *FGFR1* amplification status in hypopharyngeal and laryngeal SCC

**Fig. 1:**
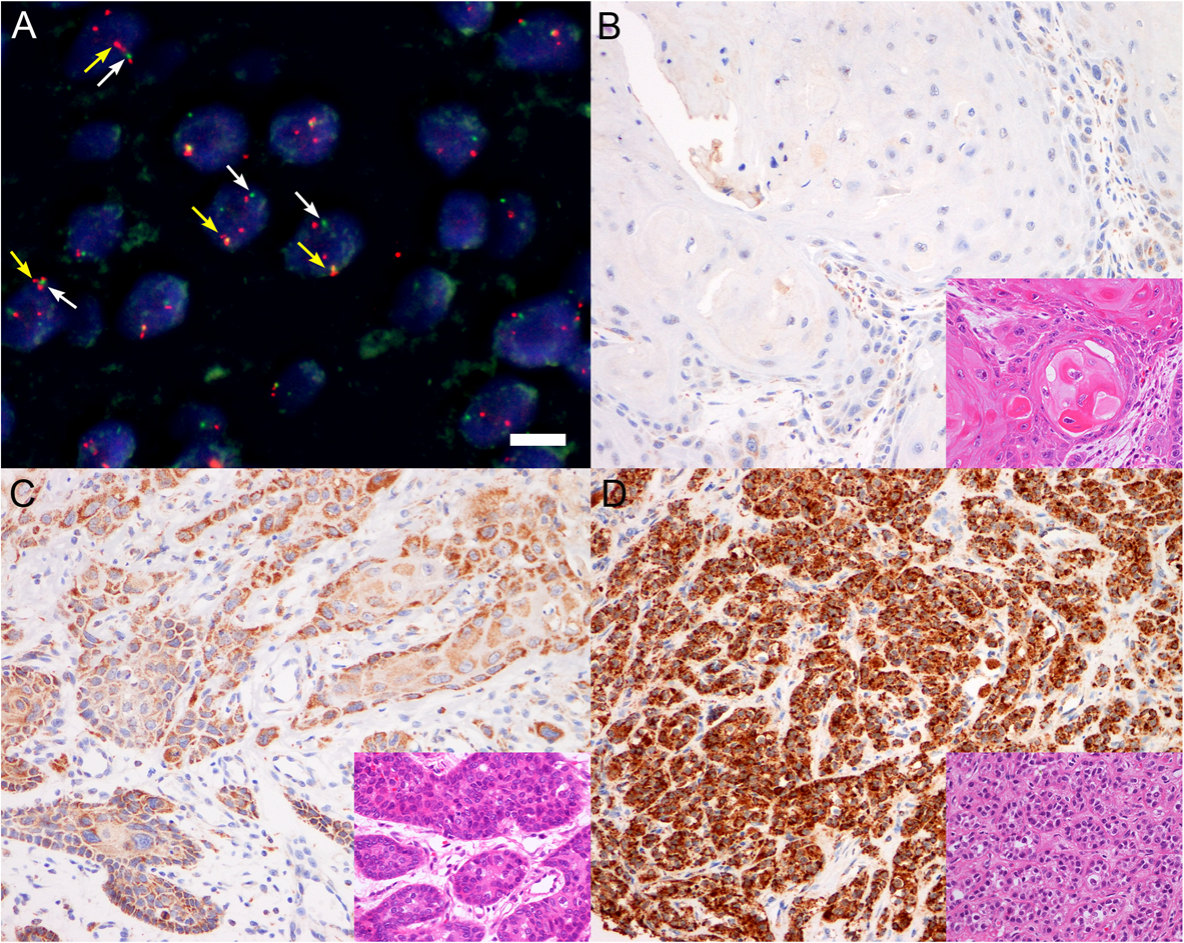
*FGFR1* FISH analysis and FGFR1 protein expression in hypopharyngeal and laryngeal squamous cell carcinoma (SCC). (**a**) Amplified *FGFR1* expression is shown as red signals (yellow arrows) and CEP8 signal (white arrows) is shown as green in nuclei. Increased red signals (in number 3~6) compared to green signal (in number 1~2) could define amplification of FGFR1 gene. Scale bar represents 10 μm. (original magnification, 1000×). Representative FGFR1 immunohistochemical staining (original magnification, 200×) showing negative (**b**), low expression (**c**), and high expression (**d**), and the corresponding hematoxylin and eosin-stained cases (inset; original magnification 400×) showing well differentiated SCC (**b**), moderately differentiated SCC (**c**), and poorly differentiated SCC (**d**)

The FISH test was finally available for 66 cases, excluding cases with decalcified tissue, insufficient amounts of tumor cells, or poor signals on FISH tests. Of these, 8 (12.1%) patients displayed *FGFR1* amplification (Table 2 and Fig. 1a). The mean *FGFR1* copy number per nucleus and the mean *FGFR1*/CEP8 ratio in all patients were 2.37 (range, 1.85 to 6.75 copies per nucleus) and 1.00 (range, 0.42 to 2.54), respectively, in 66 tested cases. The mean *FGFR1* copy number was 5.37 (range, 4.01 to 6.75) in the amplification group and 2.48 (range 1.85 to 4.86), in the non-amplification group. The mean *FGFR1*/CEP8 ratio was 2.23 (range 1.59 to 2.54) and 0.96 (range, 0.42 to 1.59) in the amplification and non-amplification group, respectively (Supplementary Fig. 1).

**Table 2:** The status of FGFR1 gene amplification and protein overexpression in patients with hypopharyngeal and laryngeal squamous cell carcinoma

Characteristics	*FGFR1* amplification, *n* = 66		*P* value	FGFR1 high expression, *n* = 199		*P* value
	Present, n (%)	Absent, n (%)		Present, n (%)	Absent, n (%)	
Total	8 (12.1)	58 (87.9)		21 (10.6)	178 (89.4)	
Primary sites			**0.015**			0.307
Hypopharynx	6 (75.0)	36 (62.1)		6 (28.6)	47 (26.4)	
Larynx	2 (25.0)	22 (37.9)		15 (71.4)	131 (73.6)	
Gender			0.771			0.145
Female	0 (0.0)	2 (3.4)		3 (14.3)	10 (5.6)	
Male	8 (100.0)	56 (96.6)		18 (85.7)	168 (94.4)	
Age (years)			0.452			0.820
Median (range)	68.0 (55–73)	63.0 (42–88)		63.0 (45–78)	64.0 (30–88)	
< 65	3 (37.5)	33 (56.9)		12 (57.1)	95 (53.4)	
≥ 65	5 (62.5)	25 (43.1)		9 (42.9)	83 (46.6)	
Smoking			0.405			0.302
Never smoker	2 (25.0)	7 (12.1)		3 (14.3)	19 (10.7)	
Former smoker	1 (12.5)	15 (25.9)		6 (28.6)	45 (25.3)	
Current smoker	5 (62.5)	36 (62.1)		12 (57.1)	114 (64.0)	
Alcohol			0.196			0.778
Non- or social- drinker	0 (0.0)	13 (22.4)		5 (23.8)	37 (20.8)	
Heavy drinker	8 (100.0)	45 (77.6)		16 (76.2)	141 (79.2)	
p16 status			0.551			0.697
Negative	7 (87.5)	47 (81.0)		14 (82.4)	131 (88.5)	
Positive	1 (12.5)	11 (19.0)		3 (17.6)	17 (11.5)	
Unknown	–					
Histologic differentiation			0.355			**< 0.001**
Well differentiated	2 (25.0)	15 (25.9)		1 (4.8)	58 (32.6)	
Moderately differentiated	4 (50.0)	37 (63.8)		11 (52.4)	108 (60.7)	
Poorly differentiated	2 (25.0)	6 (10.3)		9 (42.9)	12 (6.7)	
Lymphovascular invasion			**0.031**			0.096
Absent	4 (50.0)	50 (86.2)		13 (61.9)	141 (79.2)	
Present	4 (50.0)	8 (13.8)		8 (38.1)	37 (20.8)	
Perineural invasion			0.614			0.335
Absent	7 (87.5)	52 (89.7)		16 (76.2)	152 (85.4)	
Present	1 (12.5)	6 (10.3)		5 (23.8)	26 (14.6)	
Positive resection margin			0.537			0.494
Absent	4 (50.0)	32 (55.2)		16 (76.2)	130 (73.0)	
Present	4 (50.0)	26 (44.8)		5 (23.8)	48 (27.0)	
Pathological T-classification			0.365			0.238
pT1	3 (37.5)	29 (50.0)		4 (19.0)	59 (33.1)	
pT2	4 (50.0)	18 (31.0)		9 (42.9)	49 (27.5)	
pT3	0 (0.0)	10 (17.2)		4 (19.0)	48 (27.0)	
pT4a	1 (9.1)	1 (1.7)		4 (19.0)	22 (12.4)	
Pathological N-classification			**0.012**			**0.003**
pN0	3 (27.3)	32 (58.2)		6 (28.6)	105 (59.0)	
pN1	0 (0.0)	4 (7.3)		1 (4.8)	12 (6.7)	
pN2	1 (9.1)	7 (12.7)		4 (19.0)	22 (12.4)	
pN3	7 (63.6)	12 (21.8)		10 (47.6)	39 (21.9)	
Pathological stage			**0.047**			**0.001**
Stage I	2 (25.0)	24 (41.4)		1 (4.8)	53 (29.8)	
Stage II	0 (0.0)	5 (8.6)		3 (14.3)	26 (14.6)	
Stage III	0 (0.0)	7 (12.1)		2 (9.5)	30 (16.9)	
Stage IVA	1 (12.5)	8 (13.8)		5 (23.8)	30 (16.9)	
Stage IVB	5 (62.5)	14 (24.1)		10 (47.6)	39 (21.9)	
Recurrence			**0.042**			0.263
No	4 (50.0)	49 (84.5)		14 (66.7)	141 (79.2)	
Yes	4 (50.0)	9 (15.5)		7 (33.3)	37 (20.8)	
Local recurrence only	2 (50.0)	5 (55.6)		2 (28.6)	20 (54.1)	
Distant recurrence	2 (50.0)	4 (44.4)		5 (71.4)	17 (45.9)

The association of *FGFR1* amplification with clinical and pathological factors is summarized in Table 2. *FGFR1* amplification was more frequent in hypopharyngeal SCC than in laryngeal SCC (6/42, 14.3% versus 2/24, 8.3%; *P* = 0.015) and showed a significant correlation with the presence of lymphovascular invasion (*P* = 0.031), more advanced pathological N-classification (*P* = 0.020), more advanced TNM tumor stage (*P* = 0.047), and more frequent recurrence rate (*P* = 0.042) than cases with no *FGFR1* amplification. Other factors were not significant according to *FGFR1* amplification status. For the 8 cases with *FGFR1* amplified squamous cell carcinoma, the clinicopathologic characteristics as well as detailed status *FGFR1* gene and FGFR1 protein are summarized in Supplementary Table S1.

### FGFR1 protein expression in hypopharyngeal and laryngeal SCC

**Fig. 2:**
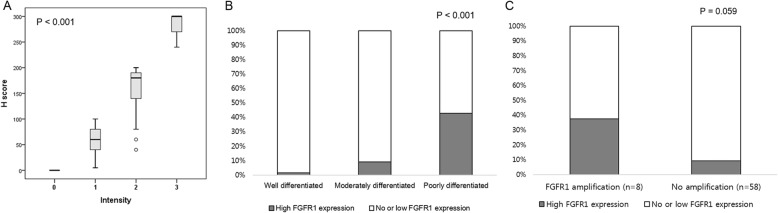
FGFR1 immunohistochemistry in hypopharyngeal and laryngeal squamous cell carcinoma. (**a**) FGFR1-positive tumor cells with strong intensity were observed in more than 80% tumor area. (**b**) High FGFR1 expression was more frequently found in poorly differentiated histology. (**c**) High FGFR1 protein expression showed a marginal tendency to be related to gene amplification

FGFR1 IHC was evaluable in 199 cases. FGFR1 staining exhibited cytoplasmic patterns with occasional weak nuclear patterns and was uniform in most tumor areas. The normal squamous epithelial cells and stromal cells adjacent to or separated from tumors were stained with an intensity from 0 to 2, and none showed strong staining with an intensity score of 3. We found cases with predominantly strong FGFR1 staining intensity (score 3) in tumor cells, wherein positive staining was observed in more than 80% tumor cells; therefore, the H-score of these cases was calculated to be more than 240 (Fig. 2a). Considering the expression pattern of FGFR1, we defined the cutoff value for high FGFR1 expression as a strong intensity and/or H-score of more than 240. The H-score of FGFR1 was only weakly correlated in stromal cells and tumor cells (r = 0.256, *P* <  0.001).

On the basis of the criteria of our series, 21 (10.6%) patients showed high FGFR1 expression. The association between FGFR1 overexpression and clinical and pathological factors is summarized in Table 2. High FGFR1 expression was more frequently detected in poorly differentiated histology (*P* <  0.001; Table 2 and Fig. 1b-d), while well differentiated SCC showed no or low FGFR1 expression (Fig. 2b). In comparison with no or low FGFR1 expression, high FGFR1 expression was associated with more advanced pathological N-classification (*P* = 0.003) and TNM stage (*P* = 0.001). Other factors were not significant according to FGFR1 expression status (Table 2). Consistent with *FGFR1* gene expression, high FGFR1 protein expression showed a marginal tendency to be related with gene amplification, although no statistical significance was observed (Fig. 2c; *P* = 0.059).

### Alteration of FGFR1 and epithelial mesenchymal transition (EMT)

Snail and Twist IHC were evaluable in 67 cases. When comparing *FGFR1* amplified SCC with non-amplified SCC, there were no statistically significant differences in Snail and Twist expression (*P* = 0.344 and *P* = 0.637, respectively; Supplementary Figure S2 and S3). The expression level of FGFR1 protein also did not correlate with the expression levels of Snail and Twist (*P* = 0.904 and *P* = 0.402, respectively; Supplementary Figure S2 and S3).

### Survival outcomes according to FGFR1 gene amplification and protein expression

**Fig. 3:**
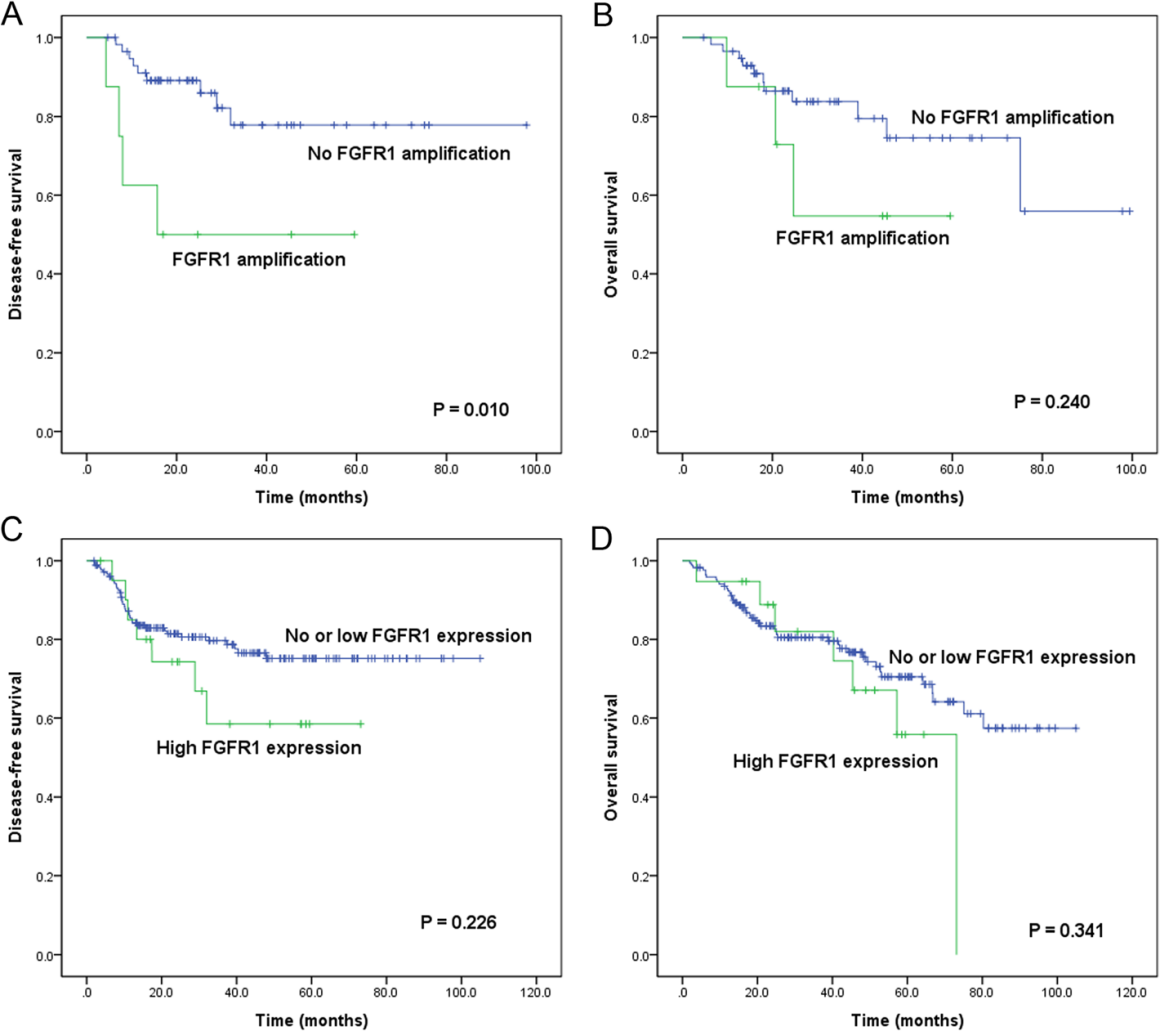
Kaplan-Meier survival curves for FGFR1 gene amplification (**a** and **b**) and protein overexpression (**c** and **d**) in hypopharyngeal and laryngeal squamous cell carcinoma. *FGFR1* amplification was significantly associated with disease-free survival (**a**) but not overall survival (**b**). FGFR1 protein overexpression was not related to disease-free survival (**c**) or overall survival (**d**)

In the Kaplan-Meir analysis, patients with *FGFR1* amplification were more associated with inferior DFS rate than those with no amplification (*P* = 0.010, Fig. 3a); however, such significant association was not observed for OS rate (*P* = 0.240, Fig. 3b). The status of FGFR1 protein expression showed no significant association with DFS and OS (*P* = 0.226 and *P* = 0.341, respectively; Fig. 3c and d).

In the univariate Cox proportional hazard analysis for DFS (Table 3), poorly differentiated histology (hazard ratio [HR] 3.803, *P* = 0.006), perineural invasion (HR 2.046, *P* = 0.039), pN-classification (N2; HR 5.415; *P* <  0.001, N3; HR 2.816; *P* = 0.005), pathologic stage (IV; HR 3.124; *P* = 0.007), and *FGFR1* amplification (HR 4.204, *P* = 0.017) were significantly related to worse DFS. Of these, *FGFR1* amplification was determined as an independent factor for poor DFS in multivariate analysis (HR 3.666, *P* = 0.049).

**Table 3:** Cox proportional hazards regression model for disease-free survival in hypopharyngeal and laryngeal squamous cell carcinoma

Variables	Univariate		Multivariate	
	*P* value	HR (95% CI)	*P* value	HR (95% CI)
≥ 65 years	0.485	1.232 (0.686–2.211)		
Male	0.961	1.030 (0.319–3.324)		
Primary sites				
Larynx		1 (reference)		
Hypopharynx	0.385	1.331 (0.698–2.536)		
Smoking				
Never smoker		1 (reference)		
Smoker	0.368	0.690 (0.308–1.546)		
Alcohol				
Non- or social- drinker		1 (reference)		
Heavy drinker	0.196	1.763 (0.746–4.164)		
Differentiation				
Well		1 (reference)		1 (reference)
Moderate	0.057	2.148 (0.979–4.715)	0.687	1.340 (0.323–5.561)
Poor	**0.006**	3.803 (1.466–9.864)	0.883	1.154 (0.170–7.839)
Lymphovascular invasion	0.120	1.669 (0.875–3.180)		
Perineural invasion	**0.039**	2.046 (1.035–4.040)	0.613	0.567 (0.063–5.119)
Positive resection margin	0.091	1.710 (0.919–3.185)		
pT-classification				
pT1		1 (reference)		
pT2	0.906	1.051 (0.463–2.384)		
pT3	0.178	1.715 (0.782–3.759)		
pT4a	0.070	2.224 (0.936–5.282)		
pN-classification				
pN0		1 (reference)		
pN1	0.062	2.881 (0.948–8.757)		
pN2	**< 0.001**	5.415 (2.492–11.769)		
pN3	**0.005**	2.816 (1.359–5.837)		
Pathological stage				
Stage I		1 (reference)		1 (reference)
Stage II	0.686	0.756 (0.195–2.929)	0.904	1.147 (0.124–10.594)
Stage III	0.168	2.089 (0.732–5.960)	0.982	< 0.001
Stage IV	**0.007**	3.124 (1.364–7.153)	0.234	2.511 (0.551–11.438)
Adjuvant treatment	0.396	1.303 (0.707–2.400)		
p16 positivity	0.791	1.073 (0.637–1.806)		
*FGFR1* amplification	**0.017**	4.204 (1.290–13.698)	**0.049**	3.666 (1.006–13.361)
FGFR1 high expression	0.235	1.633 (0.727–3.664)		

^*a*^*Abbreviations*: *CI* Confidence interval, *HR* Hazard ratio

^b^Variables of the pT and pN classification were not included in the multivariate analysis, because they were included in the pathologic stage

In the univariate Cox proportional hazard analysis for OS (Table 4), older age (≥ 65 years, HR 2.102, *P* = 0.009), primary site (hypopharynx, HR 1.812, *P* = 0.042), histologic differentiation (moderate; HR 2.885; *P* = 0.007, poor; HR 3.180, *P* = 0.026), lymphovascular invasion (HR 1.842, *P* = 0.040), perineural invasion (HR 2.608, *P* = 0.002), positive resection margin (HR 2.236, *P* = 0.006), T-classification (T3; HR 1.715; *P* = 0.032, T4a; HR 2.224; *P* = 0.011), pN-classification (N2; HR 2.982; *P* = 0.005, N3; HR 3.003; *P* = 0.001), and advanced pathologic stage (III; HR 3.890; *P* = 0.011, IV; HR 4.102; *P* = 0.004) were significantly related to inferior OS rates. Older age (≥ 65 years, HR 1.835, *P* = 0.035), positive resection margin (HR 2.712, *P* = 0.002), and advanced pathologic stage (III; HR 3.018; *P* = 0.048, IV; HR 2.967; *P* = 0.046) were identified as the independent factors for poor OS in multivariate analysis.

**Table 4:** Cox proportional hazards regression model for overall survival in hypopharyngeal and laryngeal squamous cell carcinoma

Variables	Univariate		Multivariate	
	*P* value	HR (95% CI)	*P* value	HR (95% CI)
≥ 65 years	**0.009**	2.102 (1.207–3.663)	**0.035**	1.835 (1.042–3.231)
Male	0.503	1.493 (0.463–4.816)		
Primary sites				
Larynx		1 (reference)		1 (reference)
Hypopharynx	**0.042**	1.812 (1.022–3.213)	0.702	1.142 (0.580–2.248)
Smoking				
Never smoker		1 (reference)		
Smoker	0.621	1.263 (0.500–3.190)		
Alcohol				
Non- or social- drinker		1 (reference)		
Heavy drinker	0.160	1.772 (0.798–3.937)		
Differentiation				
Well		1 (reference)		1 (reference)
Moderate	**0.007**	2.885 (1.343–6.199)	**0.033**	2.422 (1.076–5.452)
Poor	**0.026**	3.180 (1.147–8.819)	0.422	1.398 (0.532–4.513)
Lymphovascular invasion	**0.040**	1.842 (1.029–3.296)	0.569	0.819 (0.411–1.632)
Perineural invasion	**0.002**	2.608 (1.411–4.821)	0.098	1.825 (0.896–3.720)
Positive resection margin	**0.006**	2.236 (1.265–3.951)	**0.002**	2.712 (1.425–5.161)
pT-classification				
pT1		1 (reference)		
pT2	0.358	1.464 (0.649–3.301)		
pT3	**0.032**	1.715 (1.076–5.171)		
pT4a	**0.011**	2.224 (1.286–6.928)		
pN-classification				
pN0		1 (reference)		
pN1	0.085	2.385 (0.888–6.409)		
pN2	**0.005**	2.982 (1.382–6.431)		
pN3	**0.001**	3.003 (1.578–5.714)		
Pathological stage				
Stage I		1 (reference)		1 (reference)
Stage II	0.482	1.561 (0.451–5.402)	0.457	1.625 (0.452–5.847)
Stage III	**0.011**	3.890 (1.361–11.117)	**0.048**	3.018 (1.010–9.018)
Stage IV	**0.004**	4.102 (1.591–10.581)	**0.046**	2.967 (1.018–8.647)
Adjuvant treatment	0.615	1.154 (0.661–2.013)		
p16 positivity	0.127	0.331 (0.080–1.368)		
*FGFR1* amplification	0.252	2.128 (0.585–7.738)		
FGFR1 high expression	0.319	1.504 (0.674–3.354)		

^a^*Abbreviations*: *CI* Confidence interval, *HR* Hazard ratio

^b^Variables of the pT and pN classification were not included in the multivariate analysis, because they were included in the pathologic stage

## Discussion

In the present study, we investigated the clinicopathologic and prognostic implications of FGFR1 gene amplification and protein overexpression in hypopharyngeal and laryngeal SCC. We analyzed a large number of cases that underwent standard management of curative surgery and appropriate adjuvant therapy in two different institutes.

In our series, hypopharyngeal and laryngeal SCCs were generally detected in late adulthood, and frequently related to smoking and alcohol consumption and p16 IHC negativity. Furthermore, advanced tumor stages, especially advanced pathologic lymph node stages, were not uncommon, and the 5-year DFS and OS rates were 74.1 and 67.6%, respectively, indicating that a proportion of patients with this disease exhibited unfavorable outcomes. These demographic features were generally similar to those previously reported and our series may be used as a surrogate of the population-based data [30, 31]. Hypopharyngeal SCCs showed more lymph node metastasis compared to laryngeal SCC in our study and they were expected to have a worse prognosis than laryngeal SCC. However, in Kaplan-Meier analysis and multivariate Cox regression model, the difference between OS and DFS according to larynx and hypopharynx was not statistically significant. Therefore, we think combining these two groups would not cause serious selection bias, but the concept of non-HPV related SCC can be approached.

*FGFR1* gene amplification was observed in about 12% tested hypopharyngeal and laryngeal SCC cases that was slightly more frequent in hypopharyngeal SCC (14.3%) than in laryngeal SCC (8.3%). *FGFR1* amplification has been suggested as an oncogenic driver mutation in tobacco-associated cancers of the aerodigestive tract [16, 32–34]. Previous studies on *FGFR1* in HNSCC have shown that *FGFR1* amplification is more common in the SCC of hypopharynx and larynx than in that of oropharynx or oral cavity [14, 16]. In addition, *FGFR1* amplification has no relationship with HPV infection [13]. All these findings are suggestive of the biological role of *FGFR1* amplification in the tumorigenesis of these tobacco or alcohol-related cancers, hypopharyngeal and laryngeal SCCs, and may predict the role of targeted therapy for these tumors [30, 31].

Considering the clinical implications of *FGFR1* amplification, we observed significant association with poor prognostic factors, specifically, lymphovascular invasion and advanced stages of lymph node metastasis. This observation may be related to our findings that cases with *FGFR1* amplification were closely associated with advanced TNM tumor stages and poorer DFS. In particular, *FGFR1* amplification was determined as an independent factor for disease progression and thus, may be involved in the invasion, metastasis, and drug resistance of tumor cells during the development of treatment-resistant, aggressive, advanced hypopharyngeal and laryngeal SCCs. Several studies have also shown that *FGFR1* amplification plays a role in the invasion, metastasis, and drug resistance of various tumors [32, 35]. A recent study performed genomic profiling of HNSCC using targeted next-generation sequencing and identified *FGFR1* amplification as an independent prognostic factor for OS [14], while it has been failed to impact on prognosis in other studies [15, 16, 36]. In our study, we failed to detect any association between *FGFR1* amplification and OS. Cases positive for *FGFR1* amplification were relatively fewer in number in previous and present studies; therefore, its prognostic role warrants validation in future studies, including meta-analysis.

In our series, high FGFR1 expression was observed in about 11% of tested cases with hypopharynx and larynx SCCs. Similar to *FGFR1* amplification, FGFR1 protein overexpression was associated with lymph node metastasis and advanced TNM tumor stages. Considering the marginal association between high FGFR1 expression and *FGFR1* amplification, gene amplification and the subsequent protein overexpression may be one of the related mechanisms underlying the invasion and metastasis of hypopharyngeal and laryngeal SCCs. However, FGFR1 protein overexpression showed no effect on survival outcomes. Previous studies have shown a disagreement with prognostic values of FGFR1 overexpression in HNSCC, probably owing to the use of different FGFR1 overexpression criteria, anti-FGFR1 antibodies, and cohorts with different anatomical locations [13, 17, 37].

FGFR1 demonstrated strong and diffused expression in poorly differentiated SCC, while normal, dysplastic squamous epithelium, or well differentiated SCC exhibited weak to moderate expression patterns. In a recent study on oral tongue SCC, FGFR1 expression was stronger in high-grade dysplasia than in low-grade dysplasia as well as in the nucleus of poorly differentiated SCC cells [37]. However, we failed to report any increase in nuclear staining in poorly differentiated SCC. These results suggest that FGFR1 may be one of the important factors in the carcinogenesis and progression of HPV-negative, smoking- and alcohol-related SCC represented by hypopharyngeal and laryngeal SCC.

Regarding the biologic implications of FGFR1 gene amplification or protein overexpression in hypopharyngeal and laryngeal SCC, we investigated a correlation of FGFR1 alteration with twist and snail, the well-known EMT markers, on the basis of previous studies showing that EMT is induced in tumors by abnormal activation of the FGFR signaling pathway in several types of cancers including HNSCC [38–41]. However, we did not observe that amplification or high protein expression of FGFR1 is related to overexpression of these two EMT-related proteins. Therefore, further studies are needed to determine whether changes in FGFR1 affect EMT acquisition in hypopharyngeal and laryngeal SCC. In addition, further in-depth studies should be followed for the underlying mechanisms of aberrant *FGFR1* alterations in the tumorigenesis of hypopharyngeal and laryngeal SCC in the aspect of the known downstream signals of RAS/MAPK, PI3K/AKT, and JAK/STAT signaling pathways [11].

FGFR1 gene amplification and protein or mRNA expression have demonstrated correlation in some previous studies [23, 28]. However, we could not find any strong correlation between FGFR1 gene amplification and protein overexpression. This discrepancy may be associated with the differences in the cutoff level of amplification and protein or mRNA expression among various studies. Furthermore, *FGFR1* amplification may not always cause protein overexpression alone, and may be affected by other closely related receptor tyrosine kinases (RTK). In lung SCC, some researchers suggested the mechanism of discrepancy between protein expression and gene amplification of FGFR1 due to crosstalk between FGFR1 and co-activated RTKs in FGFR1-amplified lung cancers with low FGFR1 protein expression [42, 43].

## Conclusions

In summary, we report that FGFR1 gene amplification and protein overexpression occur in hypopharyngeal and laryngeal SCC with an incidence of 12.1 and 10.6%, respectively. High FGFR1 expression was more frequent with the worsening of histologic differentiation. In addition, *FGFR1* amplification appeared as an independent prognostic factor for DFS and may serve as a prognostic biomarker. These results suggest that the altered FGFR1 pathways play an important role in the malignant evolution and progression of hypopharyngeal and laryngeal SCC. Several emerging FGFR1-targeted therapies may shed light on treatment of patients with hypopharyngeal and laryngeal SCC that usually lack specific therapeutic targets.

## Supplementary information

**Additional file 1: Figure S1.** The mean *FGFR1*/CEP8 ratio (R) and the mean *FGFR1* copy number (CN) in hypopharyngeal and laryngeal squamous cell carcinoma. (A) The mean *FGFR1* R and CN were 2.37 and 1.00, respectively, in 66 tested cases. (B) The mean *FGFR1* R was 2.18 and 0.96 in the amplification and non-amplification group, respectively. (C) The mean *FGFR1* CN was 5.36 in the amplification group and 2.48, in the non-amplification group.

**Additional file 2: Figure S2.** The box-plot graphs of Snail (A and C) and Twist (B and D) immunohistochemical analysis (H-score) of protein expression in hypopharyngeal and laryngeal SCC. There is no statistically significant differences in Snail and Twist expression according to *FGFR1* amplification (*P* = 0.344 and *P* = 0.637, respectively; A and B) or high protein expression (*P* = 0.904 and *P* = 0.402, respectively; C and D).

**Additional file 3: Figure S3.** Snail (A-C) and Twist (D-F) protein expression by immunohistochemical staining in hypopharyngeal and laryngeal SCC. They both show nuclear staining. (A and D) Negative/weak, (B and E) moderate, and (C and F) strong intensities.

**Additional file 4: Table S1.** Clinicopathologic characteristics of 8 cases of *FGFR1* amplified squamous cell carcinoma.

## Data Availability

The datasets used and/or analyzed during the current study are available from the corresponding author on reasonable request.

## Abbreviations

AKT: Protein kinase B
CEP8: Chromosome 8
CI: Confidence interval
DFS: Disease-free survival
EGFR: Epidermal growth factor receptor
FGFR1: Fibroblast growth factor receptor 1
FISH: Fluorescence in situ hybridization
FFPE: Formalin-fixed, paraffin-embedded
HNSCC: Head and neck squamous cell carcinoma
HPV: Human papilloma virus
IHC: Immunohistochemistry
HR: Hazard ratio
JAK: Janus kinase
AJCC: Joint Committee on Cancer
MAPK: Mitogen-activated protein kinase
OS: Overall survival
STAT: Signal transducer and activator of transcription
SCC: Squamous cell carcinoma
TCGA: The Cancer Genome Atlas
TMA: Tissue microarray
