# Supplementary figures and images for: Prognostic implications of Fibroblast growth factor receptor 1 (FGFR1) gene amplification and protein overexpression in hypopharyngeal and laryngeal squamous cell carcinoma
Source: BMC Cancer. 2020 Apr 18;20:348. doi: 10.1186/s12885-020-06792-7 (PMC7181493; doi:10.1186/s12885-020-06792-7)

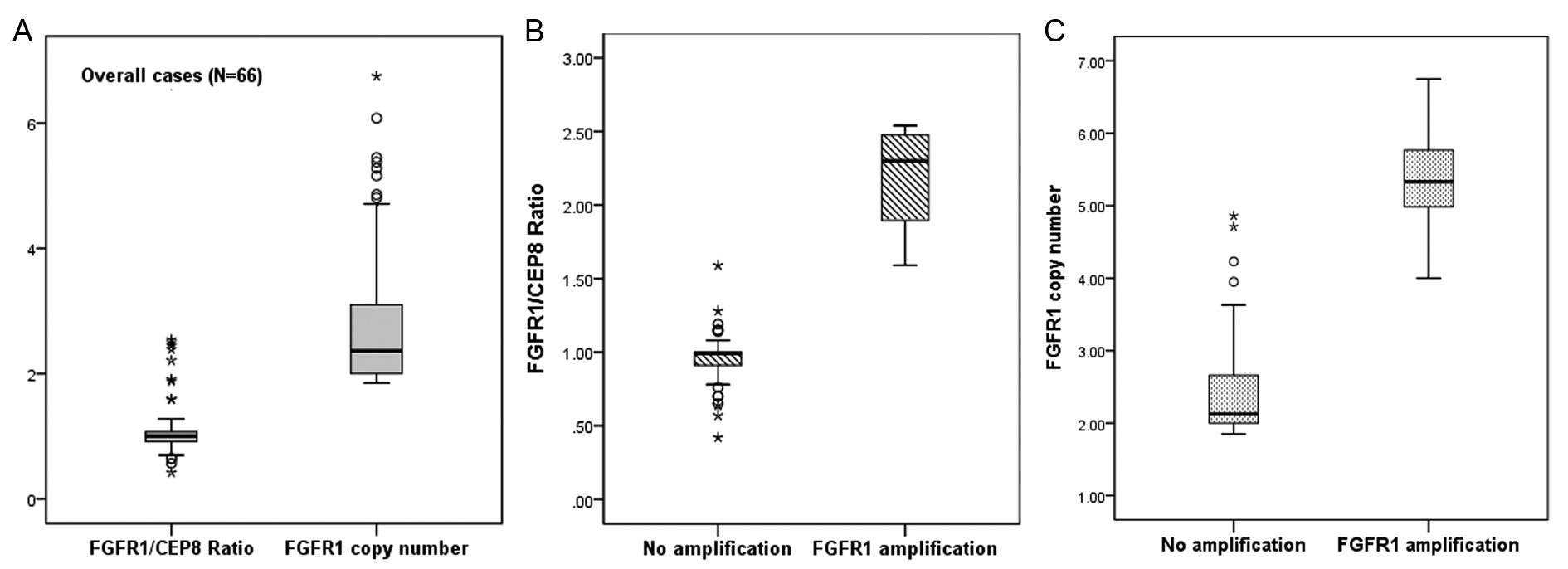

Supplement: Supplementary file 1 — Additional file 1: Figure S1. The mean FGFR1/CEP8 ratio (R) and the mean FGFR1 copy number (CN) in hypopharyngeal and laryngeal squamous cell carcinoma. (A) The mean FGFR1 R and CN were 2.37 and 1.00, respectively, in 66 tested cases. (B) The mean FGFR1 R was 2.18 and 0.96 in the amplification and non-amplification group, respectively. (C) The mean FGFR1 CN was 5.36 in the amplification group and 2.48, in the non-amplification group. [file 12885_2020_6792_MOESM1_ESM.tif]

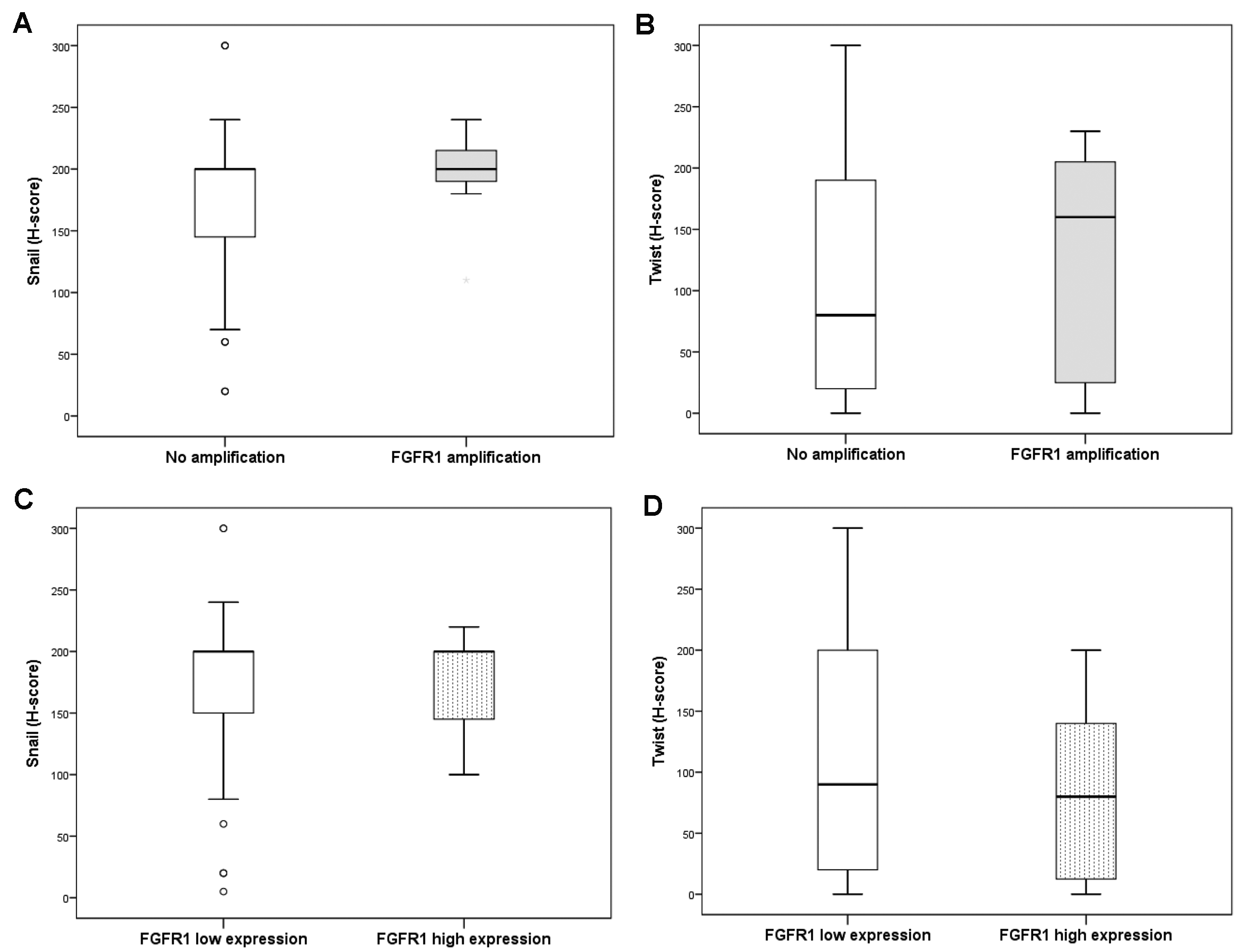

Supplement: Supplementary file 2 — Additional file 2: Figure S2. The box-plot graphs of Snail (A and C) and Twist (B and D) immunohistochemical analysis (H-score) of protein expression in hypopharyngeal and laryngeal SCC. There is no statistically significant differences in Snail and Twist expression according to FGFR1 amplification (P = 0.344 and P = 0.637, respectively; A and B) or high protein expression (P = 0.904 and P = 0.402, respectively; C and D). [file 12885_2020_6792_MOESM2_ESM.tif]

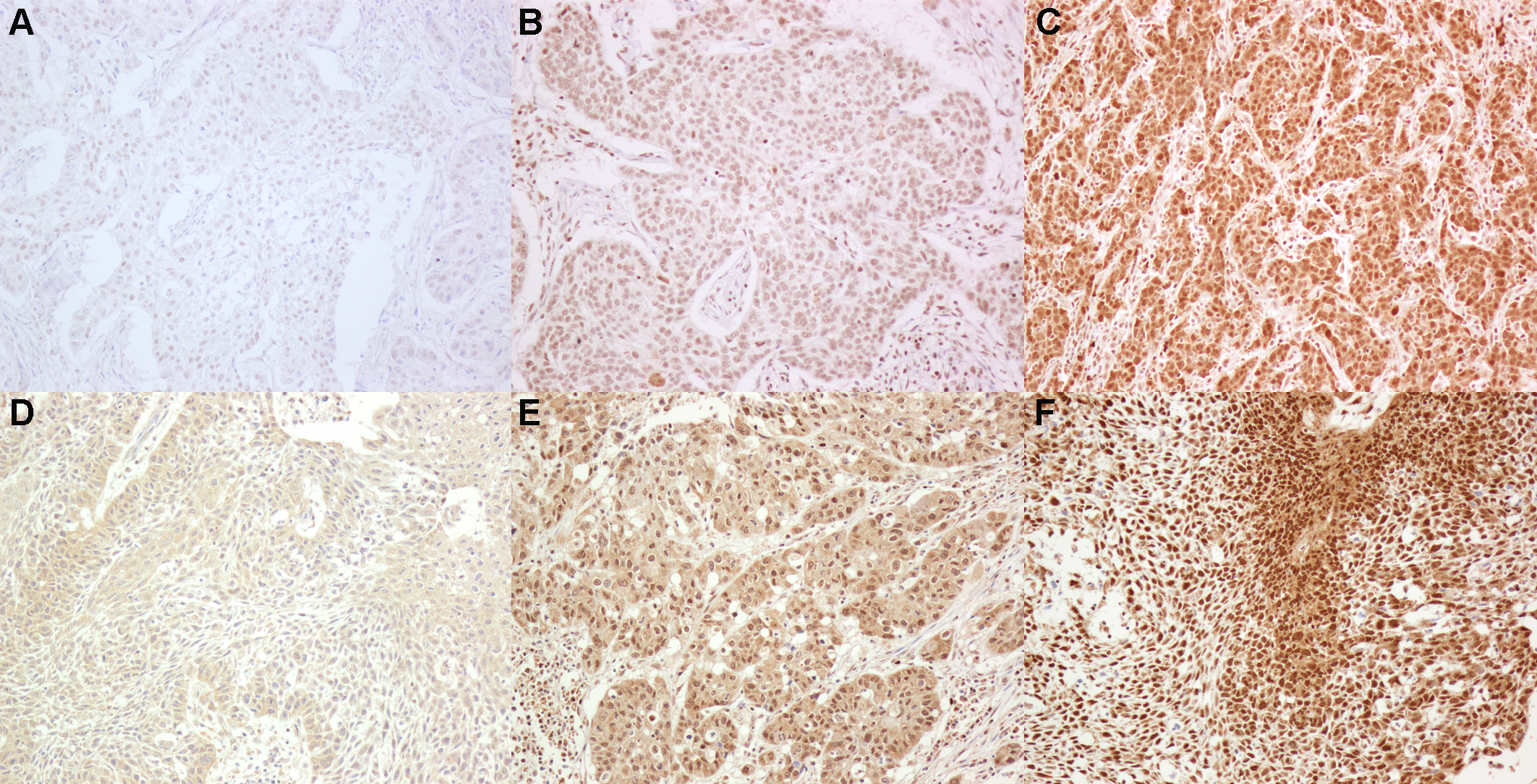

Supplement: Supplementary file 3 — Additional file 3: Figure S3. Snail (A-C) and Twist (D-F) protein expression by immunohistochemical staining in hypopharyngeal and laryngeal SCC. They both show nuclear staining. (A and D) Negative/weak, (B and E) moderate, and (C and F) strong intensities. [file 12885_2020_6792_MOESM3_ESM.tif]
