# Supplementary material for: Prognostic implications of Fibroblast growth factor receptor 1 (FGFR1) gene amplification and protein overexpression in hypopharyngeal and laryngeal squamous cell carcinoma
Source: BMC Cancer. 2020 Apr 18;20:348. doi: 10.1186/s12885-020-06792-7 (PMC7181493; doi:10.1186/s12885-020-06792-7)
Supplement: Supplementary file 4 — Additional file 4: Table S1. Clinicopathologic characteristics of 8 cases of FGFR1 amplified squamous cell carcinoma. [file 12885_2020_6792_MOESM4_ESM.docx]

**Supplementary Table S1. Clinicopathologic characteristics of 8 cases of *FGFR1* amplified squamous cell carcinoma**

| Characteristics / Case No | #101 | #160 | #14 | #120 | #132 | #142 | #145 | #172 |
| --- | --- | --- | --- | --- | --- | --- | --- | --- |
| Age (range: 55~73) |  |  |  |  |  |  |  |  |
| *FGFR1*/CEP8 ratio | 2.40 | 2.54 | 2.21 | 1.59 | 2.50 | 2.04 | 2.46 | 2.08 |
| *FGFR1* copy number | 6.75 | 5.38 | 5.17 | 6.08 | 4.01 | 5.28 | 5.45 | 4.82 |
| FGFR1 IHC (H-score) | 90 | 140 | 270 | 160 | 120 | 140 | 300 | 270 |
| Location | Larynx | Larynx | Hypopharynx | Hypopharynx | Hypopharynx | Hypopharynx | Hypopharynx | Hypopharynx |
| Histologic differentiation | Moderate | Well | Poor | Well | Poor | Moderate | Moderate | Moderate |
| Lymphovascular invasion | - | - | + | - | + | + | + | - |
| Perineural invasion | - | - | - | + | - | - | - | - |
| Smoking (current/ex-) | + | + | - | + | - | + | + | + |
| Alcohol (heavy drinker) | + | + | + | + | + | + | + | + |
| p16 status | - | - | - | - | - | - | - | + |
| pT classification | T1 | T1 | T1 | T4 | T2 | T2 | T2 | T2 |
| pN classification | N0 | N0 | N3b | N3b | N3b | N2b | N3b | N3b |
| AJCC stage | I | I | IVB | IVB | IVB | IVA | IVB | IVB |
| Recurrence | + | + | - | - | + | + | - | - |
| Survival time (months) | 44.5 | 21.0 | 59.5 | 45.5 | 9.8 | 20.7 | 24.7 | 17.0 |
